# Implementation of the trans-abdominal partial extra-peritoneal (TAPE) technique in laparoscopic lumbar hernia repair

**DOI:** 10.1186/s12893-015-0104-3

**Published:** 2015-10-28

**Authors:** Jing Sun, Xin Chen, Jianwen Li, Yun Zhang, Feng Dong, Minhua Zheng

**Affiliations:** Department of General Surgery, Ruijin Hospital, Shanghai Jiao Tong University School of Medicine, Shanghai, 200025 P.R. China; Shanghai Minimally Invasive Surgery Center, Shanghai, 200025 P.R. China

**Keywords:** Lumbar hernia, Laparoscopic repair, Trans-abdominal partial extra-peritoneal technique

## Abstract

**Background:**

There is still not any standardized operative strategy that is well-accepted all over the world for lumbarhernia. We are here to investigate the feasibility of the trans-abdominal partial extra-peritoneal (TAPE) technique in lumbar hernia repair.

**Methods:**

The TAPE technique was applied to 14 patients with lumbar hernia from May 2009 until January 2014. The surgical technique was described in details and follow-ups were performed for further evaluation.

**Results:**

The mean age of the 14 patients was 68 ± 8 years, with the average BMI 25.5 ± 2.1 kg/m^2^. The etiology study showed that 13 cases after surgical operations and one case after trauma. The average size of the hernia defect was 86.8 ± 46.4 cm^2^, while the mean size of the mesh implanted was 275 ± 61.2 cm^2^. The mean operative time was 59.2 ± 8.2 min. There was no intra-operative visceral injury in this serial of cases. There was no conversion case and all patients accepted the TAPE technique successfully. The VAS was 3.8 ± 1.9 and 2.2 ± 1.6 on POD1 and POD3, respectively. The mean post-operative hospital stay was 4.0 ± 1.3 days. The median follow-up time was 33 months. All patients returned to unrestricted movement within 2 weeks after surgery. During the follow-ups, no complication as bulge, seroma, hematoma, wound infection, abscess in surgical area and chronic pain, nor recurrence was observed.

**Conclusions:**

According to our experience in this series of investigations, the TAPE could be a feasible and easy-to-learn technique which can be applied to most of the lumbar hernia repairs.

## Background

Lumbar hernia represents a type of anatomic parietal wall defect in the lumbar region, a broad area bordered superiorly by the 12th rib, inferiorly by the iliac crest, medially by the erector spinae muscle group, and laterally by the posterior border of the external oblique muscle [1]. The incidence rate is less than 1.5 % among all the hernia cases [2]. Patients with lumbar hernia can present with pathologically flank bulge, accompanying with local discomfort and tenderness [3]. With the disease progression, severe complications such as incarceration (25 %) and strangulation (8 %) can take place and increase the operative risks [4]. Therefore, early surgical intervention is highly recommended [4]. However, due to the limitation of disease incidence and the difficulty of surgical treatment, there is still not any standardized operative strategy that is well-accepted all over the world. Nevertheless, it seemed that laparoscopic procedure has more advantages comparing to traditional open procedures [5, 6].

Recently, we applied a novel laparoscopic technique into the laparoscopic lumbar hernia repair, namely trans-abdominal partial extra-peritoneal (TAPE) technique. In this preliminary retrospective investigation, we successfully treated 14 patients with laparoscopic TAPE procedure, demonstrating the feasibility of this technique in lumbar hernia repair.

## Methods

### Patients selection

Fourteen patients diagnosed as lumbar hernia were enrolled from May 2009 until January 2014 in this retrospective study. The study was approved by clinical ethics committee of Ruijin Hospital, Shanghai Jiao Tong University School of Medicine. The written informed consents were obtained from all patients involved prior to data collection. Patients were recruited after TAPE was performed and the data was collected from retrospective chart review and postoperative follow-ups. All procedures were performed by the same surgical team. Exclusion criteria were: emergency presentation, American Society of Anesthesiologists (ASA) classification IV-V.

### Operative procedures

**Fig. 1:**
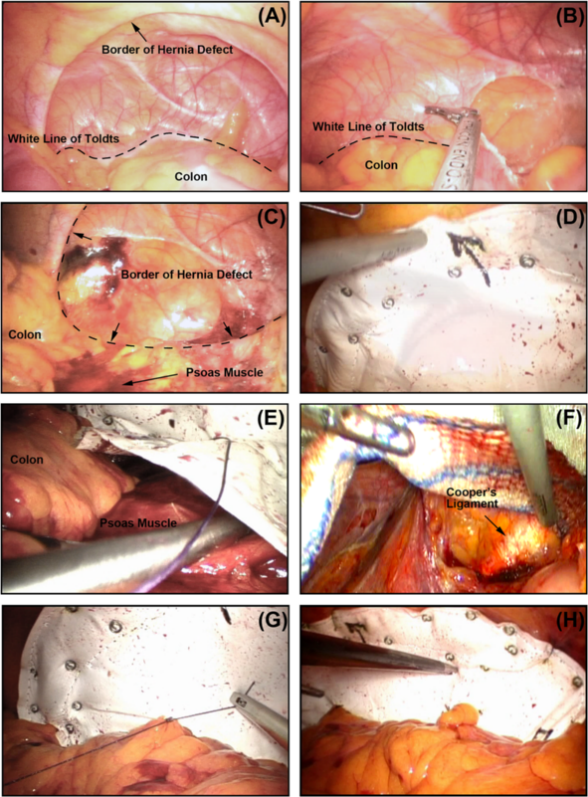
Keynotes for TAPE technique. **a** The operative field was overviewed: the white line of Toldt (dotted line) and the hernia defect was recognized; (**b**) the colon was mobilized by opening the peritoneal reflection above the white line of Toldt (dotted line); (**c**) After the colon was retracted, the extra-peritoneal space was dissected and exposed posteriorly until the psoas muscle, while the posterior border of the defect were also recognized; (**d**) A expanded polytetrafluoroethylene mesh large enough to overlap 4–5 cm onto the normal fascia was implanted and “partial-double-crown” technique was enrolled; (**e**) After overlapped with the psoas muscle, the posterior edge of the mesh was extra-peritoneally overlapped and fixed to the psoas muscle by interrupted sutures; (**f**) The dissection was extended and the mesh was fixed to the Cooper’s ligament when the inferior margin of the defect was beyond the iliac crest; (**g**) Through interrupted sutures, the colon can be restored to natural anatomical position; (**h**) An overview when finishing repair: the mesh was implanted posteriorly into extra-peritoneal space and anteriorly into peritoneal cavity, i.e., partial extra-peritoneal position

The patient was placed in a lateral decubitus position with the table flexed, opening the space between the rib cage and the iliac crest. Repair was performed under general anesthesia and pneumoperitoneum was created with a Veress technique. The abdominal pressure was maintained at 15 mmHg during the procedure. Three ports (two 5 mm and one 12 mm) were built along the middle line, with the 12 mm port located transumbilically and other two 5 mm ports located 5–6 cm away from the 12 mm port to reach both sides. Sometimes the location of trocars was different depending on the location and the size of the hernia. When the hernia is too large towards the middle line, the 12 mm port was located transrectusly on the opposite side of hernia at the umbilical level, and with other two 5 mm ports located 5–6 cm distance away from the 12 mm port to reach both sides along the vertical axis. After an overview (Fig. 1a), the colon was mobilized by opening the peritoneal reflection above the white line of Toldt (Fig. 1b). Because of gravity, the colon can be retracted so that the extra-peritoneal space can be dissected and exposed. Continuing with the mobilization posteriorly until the psoas muscle (Fig. 1c). Beware of the extra-peritoneal structure, e.g. ureter, vessels and nerves, etc. The borders of the defect were recognized before dissection was extended (Fig. 1c). Usually, the dissection was extend to the iliac bone inferiorly and 4–5 cm over the border of the defect superiorly. An expanded polytetrafluoroethylene mesh (COMPOSIX^™^ E/X, BARD DAVOL Inc., Warwick, RI, USA; PROCEED™, Ethicon Inc., Somerville, NJ, USA) large enough to overlap onto the normal fascia was inserted via the 12 mm trocar and extended close to the defect. The “partial-double-crown” technique [7] was enrolled: by 5 mm helical tack (ProTack, Tyco, Covidien, Norwalk, CT, USA), the mesh were fixed anteriorly to the musculus transverses abdominis, superiorly to subcostal ventral wall, inferiorly to iliac bone; another row of tacks was applied over the mesh around the margins of the hernia defect (Fig. 1d). After overlapped with the psoas muscle, the posterior edge of the mesh was extra-peritoneally fixed to the psoas muscle by interrupted sutures (Fig. 1e) and/or synthetic cyanoacrylic glue (COMPONT, Beijing Compont Medical Devices CO.,LTD., Beijing, P.R. China). In case the inferior margin of the defect was beyond the iliac crest, the dissection was extended and the mesh was fixed to the Cooper’s ligament (Fig. 1f). On the other hand, if the superior margin of the defect was close to subcostal margin, the mesh was implanted with at least 5 cm overlap to the diaphragm, avoiding fixation to the diaphragm to prevent associated cardiovascular and pulmonary complications. When fixation was finished, the mesh can be anatomically considered as the peritoneum. Therefore, the colon can be restored to natural anatomical position through interrupted sutures to the mesh, making use of the reserved peritoneum above the white line of Toldt (Fig. 1g). The gravity effect from the colon can stabilize the mesh in the early postoperative stage. When finishing the repair, the mesh was implanted posteriorly into extra-peritoneal space and anteriorly into peritoneal cavity, *i.e.*, partial extra-peritoneal position (Fig. 1g). Finally, the cavity was reviewed (Fig. 1h), all trocars were removed and the abdomen was deflated.

### Perioperative evaluation and postoperative follow-up

Demographic and operative data were obtained regarding age, gender, BMI (Body Mass Index, kg/m^2^), ASA score, concomitant disorders, history of previous abdominal surgery and/or trauma, hernia defect size, mesh size, operative time (defined as skin-to-skin time), blood loss, intra-operative intra-abdominal structure injury (including spleen, ureter, bowel, vessel and nerve injuries) and conversion. Perioperative data including analgesia usage, Visual Analog Scales (VAS) score on POD1 (postoperative day 1) and POD3 (postoperative day 3), transient paraesthesia, postoperative duration of hospital stay were recorded.

All patients were followed-up after being discharged, according to a pre-established protocol. This included recording of medical history as well as physical examination, which were assessed 1, 3, 6 months after surgery and every 1 year thereafter. CT scan of the abdomen was performed 1 year after surgery or when necessary. Time of returning to unrestricted movement was also recorded. Postoperative complications were evaluated as follows: seroma, hematoma, wound infection, abscess in surgical region, chronic pain, bulge, mesh displacement and recurrence. The last date for follow-ups was 31th, July 2015.

### Statistical analysis

Data are expressed as mean ± standard deviation. All calculations were performed with the SPSS software package version 12.0 (SPSS Inc., Chicago, IL, USA).

## Results

### Demographic results

Fourteen patients were enrolled in this study (Table 1). The mean age of the patients was 68 ± 8 years. Twelve patients enrolled were male while two enrolled were female, with the average BMI 25.5 ± 2.1 kg/m^2^. All patients were diagnosed as unilateral lumbar hernia, within 10 locating on left side and four on right side. The etiology study showed that 13 lumbar hernia occurred after surgical operations and one case after trauma.

**Table 1:** Demographic data

Demographic data	Patients enrolled (*N* = 14)
Age (year, mean ± SD)	68 ± 8
Gender (n/%)	
Male	12/85.7
Female	2/14.3
Body mass index (kg/m^2^, mean ± SD)	25.5 ± 2.1
Hernia side type (n/%)	
Left flank	10/71.4
Right flank	4/28.6
Etiology (n/%)	
Surgical	13/92.9
Traumatic	17.7
Concomitant disorders (n/%)	3^a^/21.4

^a^2 cases were diagnosed as lumbar hernia combined with inguinal hernia of the same side. Therefore, this patient accepted the TAPE technique after a TAPP repair for the inguinal hernia; 1 case was diagnosed with retroperitoneal cyst and accepted the TAPE technique after a cystectomy. The operative time excluded the time spent on the extra surgery

### Perioperative outcomes

As the results showed in Table 2, intra-operative investigation concluded that the average size of the hernia defect was 86.8 ± 46.4 cm^2^, while the mean size of the mesh implanted was 275 ± 61.2 cm^2^. The mean operative time was 59.2 ± 8.2 min. There was no intra-operative visceral injury in this serial of cases. There was no conversion case and all patients accepted the TAPE technique successfully. No patients accepted postoperative analgesia and the average VAS score on POD1 and POD3 was 3.8 ± 1.9 and 2.2 ± 1.6, respectively. The mean postoperative hospital stay was 4.0 ± 1.3 days.

**Table 2:** Perioperative parameters

Perioperative parameters	Patients enrolled (*N* = 14)
Hernia size (cm^2^, mean ± SD)	86.8 ± 46.4
Mesh size (cm^2^, mean ± SD)	275 ± 61.2
Operative time (min, mean ± SD)	59.2 ± 8.2
Intra-operative visceral injury (n/%)	0/0
Conversion (n/%)	0/0
Analgesia usage (n/%)	
Involved	0/0
Not-involved	14/100
VAS/POD1 (mean ± SD, 1–10)	3.8 ± 1.9
VAS/POD3 (mean ± SD, 1–10)	2.2 ± 1.6
Postoperative hospital stay (day, mean ± SD)	4.0 ± 1.3

### Follow-up results

No patient lost to follow-up during the investigation. The median follow-up time was 33 months. During the follow-ups, no complications as seroma, hematoma, wound infection, abscess in surgical area and chronic pain were observed. The follow-ups showed that all the patients returned to unrestricted movement within 2 weeks after surgery. There was no recurrence occurred according to postoperative physical examinations and/or CT scan during follow-ups.

## Discussion

It has been argued for years about the optimal surgical procedure since the concept of lumbar hernia has been raised, due to its relative rarity [8–10]. For open repair, a large incision is usually required because palpation inadequately defines the external defect [9]. In addition, the surrounding bony structure may become one of the obstacles for the exposure and adequate mesh overlap and fixation [8]. Moreover, subcostal nerve can be transected or injured by the lateral lumbodorsal fascia1 incision, which is likely to cause the muscular weakness and a subsequent larger lumbar hernia [11]. Therefore, the conventional mesh repair cannot be regarded as a perfect procedure for lumbar hernia repair. Nevertheless, all open repair techniques conclude a significant postoperative morbidity and long postoperative recovery, caused by extensive dissection during these procedures [3, 8, 12, 13].

With the development of laparoscopic techniques, laparoscopic approach appears to have lots of advantages when applied to ventral hernia repair [8, 14]. Trans-abdominal pre-peritoneal (TAPP), totally extra-peritoneal (TEP) and intra-peritoneal onlay mesh (IPOM) technique, which are major laparoscopic approaches for lumbar hernia, have significant advantages [8, 14]. Laparoscopic procedure explodes exact location of the anatomic defect, provides an excellent anatomic view, prevents injury to abdominal contents (spleen, ureter, vessels and nerves, etc.) and avoids extensive exploration and/or dissection of the lumbar region through large incision [2, 15]. Furthermore, laparoscopic approach can help to discover and treat the missed or concealed hernia during preoperative evaluations [8]. Moreover, the mesh can be stabilized since it is placed in the deep layer of abdominal wall, supported by intra-abdominal pressure [3]. In addition, the well-known advantages of the laparoscopic approach (less postoperative pain, hospital stay and wound infection, as well as faster recovery, better cosmetic appearance and minimal influence to daily life) [9] further support the involvement of laparoscopic techniques into lumbar hernia repair. Therefore, recently more specialists prefer to use laparoscopic techniques for lumbar hernia, considering that the techniques has conquered the disadvantages of open repair together with the better clinical outcomes.

However, traditional laparoscopic procedures, such as TAPP, TEP and IPOM, also have certain limitations when applied to lumbar hernia repair. Considering that most of the lumbar hernia appears to be large in size and the defect has been expanded posteriorly over the white line of Toldt towards the extra-peritoneal space behind colon, which are the key technical challenge for repairing the posterior defect of the hernia behind the colon. Therefore, the IPOM technique could not be applied directly into lumbar hernia repair. Some surgeons enrolled a modified IPOM technique during which the colon was mobilized but was left down and not secured to the mesh, while the mesh was posteriorly overlapped but not fixed towards the psoas muscle [14]. However, the mobilization of colon leading to unphysiological anatomical characteristic, while the lack of the mesh fixation towards the psoas muscle diminishing the relative strength of the posterior repair, are the inadequacies of this modified IPOM technique. The TEP technique does not imply the violation of the peritoneal cavity but acquires the total extra-peritoneal procedure. With the difficulty of total extra-peritoneal procedure due to scar tissue formation and severe adhesion caused by surgery and/or trauma, as well as long learning curve, it is not extensively used. Only a few publications referring to this technique have been reported for the treatment of lumbar hernia [16–18]. It is also concluded that an extra-peritoneal access might only be feasible for small hernias with no history of surgery (congenital or traumatic hernias) [19], which means for lumbar hernia, that is generally large or there is a history of previous surgery (secondary incision), the intra-peritoneal approach may be the safer and more advisable [19]. Moreover, when referring to TAPP procedure, according to the literature review, the size of the lumbar hernia defect of the cases reported are usually small (1.5 ~ 4 cm) [6, 14, 20]. However, for the cases with larger defect, it may be difficult for TAPP to achieve enough extraperitoneal space superiorly and inferiorly because of regional bony structures. Moreover, for those secondary lumbar hernia cases with surgical and/or traumatic lacerated peritoneum, more difficulties will be taken place for re-peritonealization. Not to mention that it is hard to ensure the implanted mesh is completely covered by the peritoneum to prevent postoperative complications. What’s more, the tearing of the peritoneum due to over-dissection and/or improper dissection of extra-peritoneal space may lead to failure of re-peritonealization.

Considering of these inadequacies above, innovations and technical revolutions have been made for laparoscopic lumbar hernia repair. Palanivelu C, et al. reported their preliminary explorations applying a combined suture and “double-mesh” technique into lumbar hernia repair [21]. Sharma A, et al. first reported the treatment of suprasymphysis hernia by a novel technique which was named as TAPE [7]. Considering the similarity of the suprasymphysis hernia and lumbar hernia, i.e., they are both boundary ventral hernias that could have been hindered by certain visceral organs (bladder, colon, etc.) and/or bony structures (symphysis joint, iliac crest, etc.) during the laparoscopic repair, we also named our technique TAPE technique for lumbar hernia repair. The TAPE technique can be regarded as the hybrid approach of both IPOM and TAPP techniques. Moreover, we also made several improvements comparing to these previous reports. In our TAPE procedure, the mesh was posteriorly overlapped and well-fixed to psoas muscle. We performed interrupted sutures for our early cases. Concerning to the risk of nerve, muscle and vessel damage caused by suture, we improved the technique and use the synthetic glue instead in the following cases. The restoration of the colon can provide a natural anatomical position of intra-abdominal organs. More importantly, the gravity effect from the restored colon together with the suture/glue fixation can stabilize the mesh and strengthen the posterior ventral wall. Therefore, our TAPE technique simplifies the surgical procedure and reduces the operative difficulty.

## Conclusion

In conclusion, TAPE technique restores the natural anatomical position of the colon easily by interrupted sutures, strengthening the posterior ventral wall. This technique can diminish the dissection and exploration of the extra-peritoneal space with less invasiveness, while enough overlap for the mesh onto the normal fascia in all directions can be easily reached. In addition, in our study, no complications or recurrence were observed after long-term follow-ups. Therefore, according to our experience in this series of investigations, the TAPE could be a feasible and easy-to-learn technique which can be applied to most of the lumbar hernia repairs.

## Abbreviations

ASA: American Society of Anesthesiologists
BMI: Body mass index
POD: Post-operative day
TAPE: Trans-abdominal partial extra-peritoneal
TAPP: Trans-abdominal pre-peritoneal
TEP: Totally extra-peritoneal
IPOM: Intra-peritoneal onlay mesh
VAS: Visual analog scales
